# Reference gene selection for expression studies in the reproductive axis tissues of Magang geese at different reproductive stages under light treatment

**DOI:** 10.1038/s41598-021-87169-z

**Published:** 2021-04-07

**Authors:** Bei-Bei Zhang, Xu Shen, Xiu-Jin Li, Yun-Bo Tian, Hong-Jia Ouyang, Yun-Mao Huang

**Affiliations:** Guangdong Provincial Key Laboratory of Waterfowl Healthy Breeding, College of Animal Science & Technology, Zhongkai University of Agriculture and Engineering, Guangzhou, 510225 Guangdong Province China

**Keywords:** Gene expression analysis, Animal physiology

## Abstract

In quantitative PCR research, appropriate reference genes are key to determining accurate mRNA expression levels. In order to screen the reference genes suitable for detecting gene expression in tissues of the reproductive axis, a total of 420 (males and females = 1:5) 3-year-old Magang geese were selected and subjected to light treatment. The hypothalamus, pituitary and testicular tissues were subsequently collected at different stages. Ten genes including *HPRT1*, *GAPDH*, *ACTB*, *LDHA*, *SDHA*, *B2M*, *TUBB4*, *TFRC*, *RPS2* and *RPL4* were selected as candidate reference genes. The expression of these genes in goose reproductive axis tissues was detected by real-time fluorescent quantitative PCR. The ΔCT, geNorm, NormFinder and BestKeeper algorithms were applied to sort gene expression according to stability. The results showed that *ACTB* and *TUBB4* were the most suitable reference genes for the hypothalamic tissue of Magang goose in the three breeding stages; *HPRT1* and *RPL4* for pituitary tissue; and *HPRT1* and *LDHA* for testicular tissue. For all three reproductive axis tissues, *ACTB* was the most suitable reference gene, whereas the least stable reference gene was *GAPDH*. Altogether, these results can provide references for tissue expression studies in geese under light treatment.

## Introduction

The reverse transcription quantitative real-time polymerase chain reaction (RT-qPCR) is a method of quantitative analysis using a starting template and evaluating the relationship between cycle threshold (Ct) values and a standard curve^1^. Given its rapidity, sensitivity, specificity and high flux, this method has been recognized as the benchmark approach for quantify and validate gene expression. However, the RNA yield, quality, reverse transcription efficiency, and PCR amplification efficiency among different samples all affect the accuracy of quantitative analysis; thus, introduction of the stable expression of reference genes for normalization is necessary^2,3^.

Stable reference genes are the key to accurate quantification of gene expression^4,5^. An ideal reference gene would show stable expression for a particular tissue type, independent of the developmental stage or experimental treatment conditions. Such as *ACTB* and *GAPDH* are widely used as common reference genes. However, a large number of studies have shown that the expression of these genes may not be consistent under various experimental conditions and in various tissues under investigation^6–8^. Therefore, it is better to screen appropriate reference genes in expression research, especially under specific experimental treatment conditions.

At present, many mathematical algorithms have been developed to facilitate the identification of stable internal reference genes, including the geNorm, NormFinder and BestKeeper programs^4,9,10^. Many studies have reported the evaluation of reference genes in different tissues of poultry at different physiological stages. Carlos et al*.*^11^ reported that *HMBS* and *HPRT1* are the most stable reference genes in the pectoralis major muscle of 1-day-old chicken. Caroline et al*.*^12^ identified 10 genes from the pectoral muscle of broilers, among which *RPL30* and *RPL5* were the most stable genes. Zhang et al*.*^13^ studied the expression stability of four candidate internal reference genes in different tissues of 120-day-old Hy-line brown layer hens, and showed that *RPS2* and *β-actin* genes had the highest relative stability. During the breeding period in geese, *GAPDH* is relatively stable both in the egg laying stage and before the onset of lay, while *18S* is relatively stable before the onset of lay and shows poor stability in the early egg laying stage^14^. In different tissues of the white king pigeon, the internal reference gene combination of *RPS2* + *18S* rRNA was found as most suitable for gene expression studies^15^. However, the systematic evaluation of reference genes in geese is still rare, especially in those subjected to light treatment. In this study, four algorithms were used to rank the applicability of 10 common reference genes in hypothalamus, pituitary and testis tissues of Magang geese under light treatmen. The results are expected to provide a reference for gene expression research in reproductive axis tissue of geese under light treatment.

## Results

### Primers specificity and efficiency

**Figure 1:**
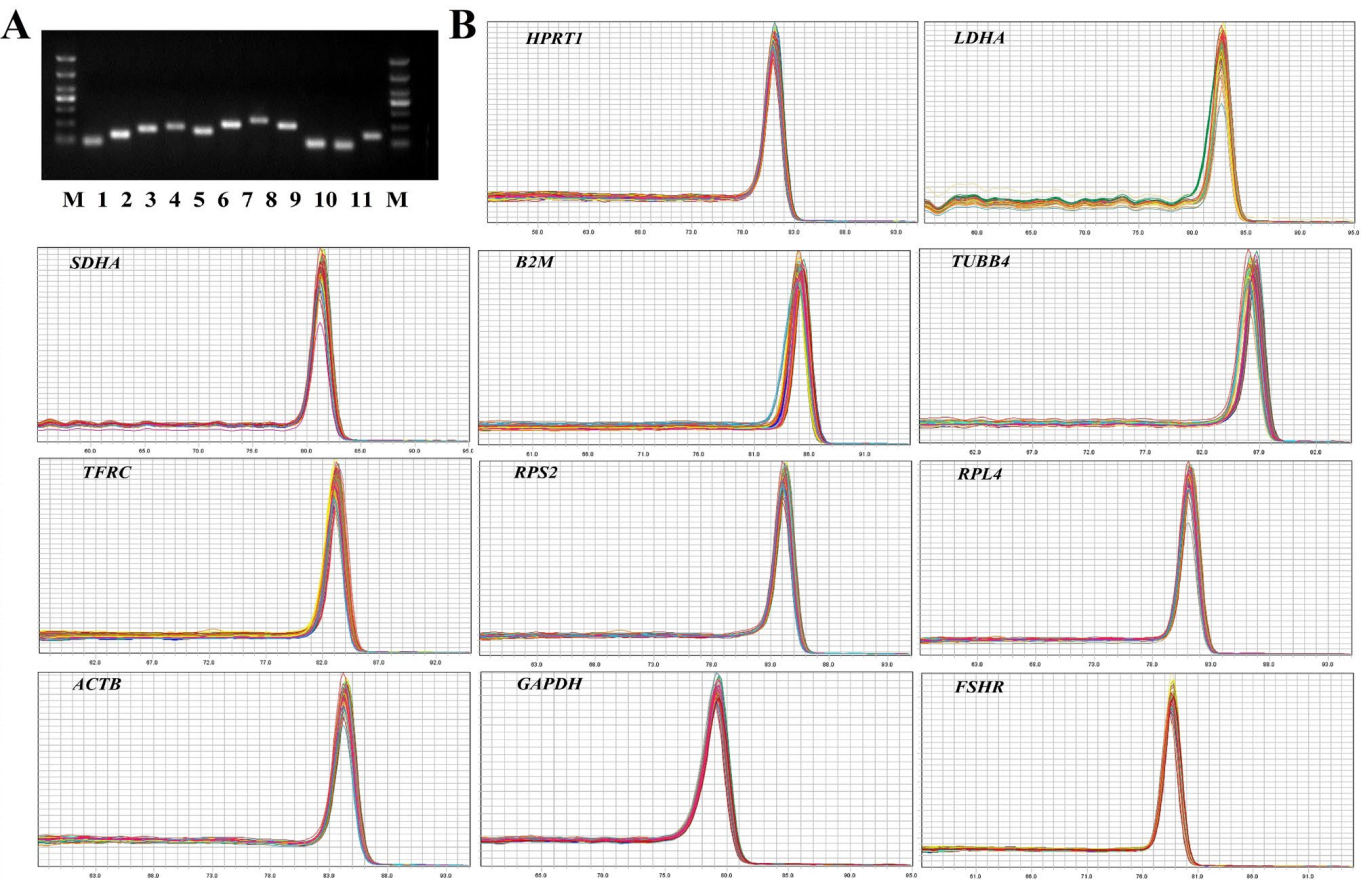
Primer specific detection of 10 candidate reference genes and verification gene FSHR. (**A**) PCR agarose gel electrophoresis; (**B**) Melting curves. M: DL1000 DNA marker; 1: *HPRT1*; 2: *LDHA*; 3: *SDHA*; 4: *B2M*; 5: *TUBB4*; 6: *TFRC*; 7: *RPS2*; 8: *RPL4*; 9: *ACTB*; 10: *GAPDH*; 11: *FSHR*.

A total of 10 candidate genes were evaluated as potential reference genes in this study. The annealing temperature of each of the primers was confirmed by gradient PCR (Table 1). Specificity of each of the primers was confirmed by gel electrophoresis and dissociation curve analysis at the expected primer annealing temperature. All primers showed amplification in gel electrophoresis as single bands with the expected amplicon sizes (Fig. 1A), and single peaks in the melting curve analysis, confirming the specificity of the primer pairs (Fig. 1B).

**Table 1:** Annealing temperature and qPCR efficiency of 10 candidate reference genes.

No	Gene symbol	Ta (°C)	Amplicon size (bp)	Efficiency (%)	Correlation coefficient (R^2^)	Slope
1	HPRT1	53.0	112	107.615	0.985	− 3.152
2	LDHA	55.0	124	100.361	0.990	− 3.313
3	SDHA	55.0	159	97.513	0.998	− 3.383
4	B2M	56.0	175	98.149	0.990	− 3.367
5	TUBB4	57.0	208	94.980	0.982	− 3.448
6	TFRC	57.0	206	107.117	0.997	− 3.162
7	RPS2	58.0	224	99.499	0.994	− 3.334
8	RPL4	58.0	198	95.366	0.986	− 3.438
9	ACTB	58.0	92	92.845	0.997	− 3.506
10	GAPDH	58.0	96	99.009	0.997	− 3.346

*Ta* annealing temperature.

To construct the standard curve, the logarithm of the copy number was considered the abscissa, and the Ct value was considered the ordinate. The efficiency of the primers ranged from 92.85 to 107.62% and R^2^ > 0.98 (Table 1).

### Expression levels of candidate reference genes

**Figure 2:**
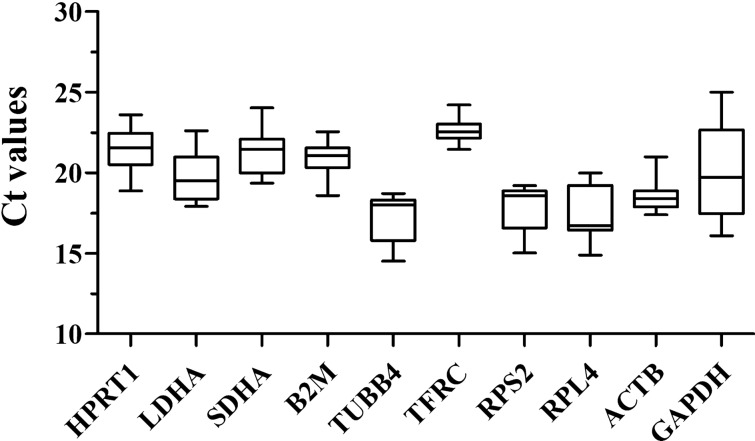
Cycle threshold (Ct) variation in tissues of the reproductive axis of Magang geese during different reproductive stages.

For each candidate reference gene, Ct values were presented for the expression data from reproductive axis tissues. The results showed variability among the various reference candidate genes, with mean Ct values ranging from 15 to 25 (Fig. 2). The expression of *GAPDH* showed wide variation among different tissues, whereas the expression of *ACTB* was relatively high and concentrated among different tissues.

### Determination of expression stability of reference candidate genes

**Figure 3:**
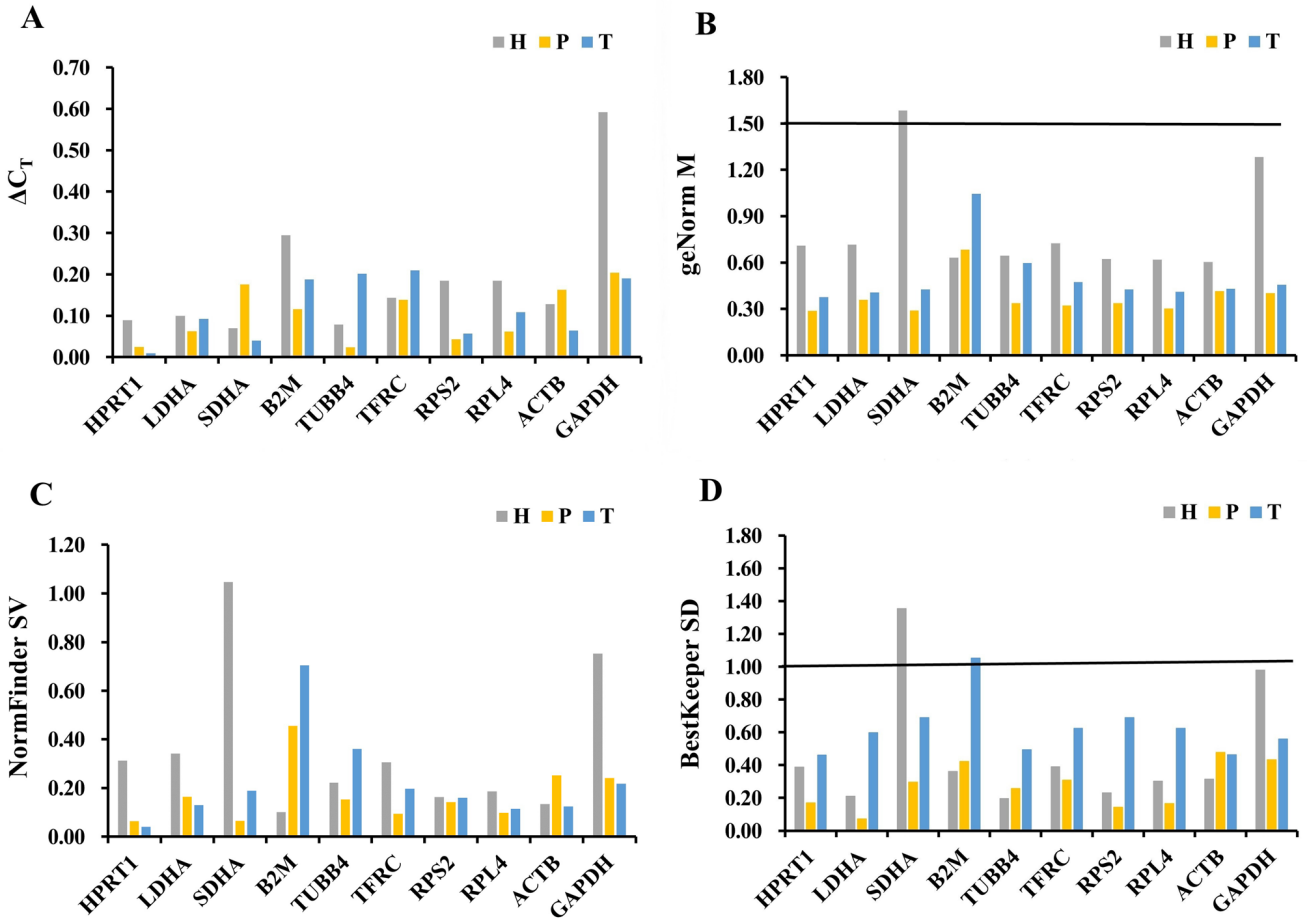
Analysis of reference gene stability based on different algorithms. (**A**) ΔCt; (**B**) geNorm; (**C**) NormFinder; (**D**) BestKeeper. *H* hypothalamus, *P* pituitary, *T* testis, *M* Average expression stability, *SV* stability value, *SD* standard deviation.

Stability in expression of the 10 candidate reference genes was determined using the mathematical algorithms Comparative ΔCT, geNorm, NormFinder and BestKeeper. The results of the ΔCT method showed that the top two genes in the hypothalamic tissue of the Magang goose in the three breeding states were *LDHA* and *TFRC*; in pituitary tissue, *TUBB4* and *HPRT1*; and in testicular tissue, *HPRT1* and *SDHA* (Fig. 3A). The geNorm ranks genes according to their average expression stability (M). The more stable the expression, the lower the M value. To identify stable reference genes, the threshold of the geNorm M value was set to 1.5. With the exception of the stable value of *SDHA* gene in hypothalamic tissue, all genes displayed stability values below the accepted threshold. The results of the geNorm analysis showed that the top two genes in hypothalamic tissue of the Magang goose in three breeding stages were *ACTB* and *RPL4*; in pituitary tissue, *SDHA* and *HPRT1*; and in testicular tissue, *LDHA* and *HPRT1* (Fig. 3B). The results of the NormFinder analysis were generally consistent with those of the geNorm analysis. However, the only exceptions in the NormFinder analysis were the second-ranked genes in hypothalamic and pituitary tissue, which were *B2M* and *RPS2*, respectively (Fig. 3C). In BestKeeper analysis, the best reference genes were those that had the lowest coefficient of variance and standard deviation (SD). Reference genes with a SD value < 1 are considered stable. The results showed that the *SDHA* gene SD > 1 in hypothalamic tissue did not meet the criteria for internal reference gene screening; thus, subsequent analysis was not performed. The top two genes in the hypothalamic tissue at the three breeding stages were *LDHA* and *TUBB4*; in pituitary tissue, *LDHA* and *RPS2*; and in testis tissue, *ACTB* and *HPRT1* (Fig. 3D).

The results of the four algorithms enabled a comprehensive analysis, and the ranking of 10 candidate reference genes are summarized in Table 2. Among the reproductive axis tissues of Magang geese at different reproductive stages, the reference genes most suitable for hypothalamic tissues were *ACTB* and *TUBB4*, and the least stable reference genes were *GAPDH* and *SDHA*. As reference genes, both *HPRT1* and *RPL4* showed good stability in pituitary tissues, whereas *ACTB* and *GAPDH* showed poor stability. In testicular tissue, the most stable internal reference genes were *HPRT1* and *LDHA*, whereas *B2M* and *TFRC* were less stable. Among all three tissues, *ACTB* was the most stable reference gene, and *GAPDH* was the least stable.

**Table 2:** Expression stability analysis of each candidate reference gene for the reproductive axis tissues based on ΔCt, geNorm, NormFinder and BestKeeper algorithms.

Group	Gene symbol	ΔCt		geNorm		NormFinder		Bestkeeper	
		Rank	ΔCt value	Rank	M value	Rank	SV value	Rank	SD
ALL	HPRT1	3	0.043	9	2.100	9	1.344	4	0.941
	LDHA	1	0.020	7	1.586	7	0.773	6	1.055
	SDHA	6	0.071	5	1.436	2	0.527	5	0.942
	B2M	8	0.090	2	1.407	3	0.538	3	0.717
	TUBB4	9	0.091	3	1.409	4	0.558	7	1.290
	TFRC	2	0.040	6	1.507	6	0.734	2	0.459
	RPS2	7	0.078	4	1.421	5	0.578	8	1.354
	RPL4	5	0.062	8	1.684	8	0.868	9	1.447
	ACTB	4	0.050	1	1.304	1	0.363	1	0.445
	GAPDH	10	0.228	10	2.300	10	1.465	10	2.098
H	HPRT1	3	0.089	6	0.709	7	0.312	7	0.388
	LDHA	4	0.100	7	0.715	8	0.340	2	0.211
	SDHA	1	0.070	10	1.584	10	1.046	10	1.354
	B2M	9	0.294	4	0.632	1	0.101	6	0.362
	TUBB4	2	0.079	5	0.644	5	0.222	1	0.197
	TFRC	6	0.143	8	0.725	6	0.305	8	0.391
	RPS2	8	0.185	3	0.623	3	0.163	3	0.231
	RPL4	7	0.184	2	0.618	4	0.185	4	0.303
	ACTB	5	0.128	1	0.603	2	0.133	5	0.316
	GAPDH	10	0.591	9	1.283	9	0.752	9	0.979
P	HPRT1	2	0.024	1	0.288	1	0.063	4	0.171
	LDHA	5	0.062	7	0.358	7	0.163	1	0.073
	SDHA	9	0.175	2	0.289	2	0.065	6	0.296
	B2M	6	0.116	10	0.683	10	0.455	8	0.423
	TUBB4	1	0.024	5	0.337	6	0.153	5	0.259
	TFRC	7	0.138	4	0.323	3	0.093	7	0.309
	RPS2	3	0.043	6	0.337	5	0.141	2	0.144
	RPL4	4	0.062	3	0.303	4	0.098	3	0.166
	ACTB	8	0.163	9	0.415	9	0.251	10	0.479
	GAPDH	10	0.204	8	0.401	8	0.240	9	0.434
T	HPRT1	1	0.009	1	0.377	1	0.040	1	0.461
	LDHA	5	0.092	2	0.406	4	0.129	5	0.598
	SDHA	2	0.040	4	0.426	6	0.189	8	0.690
	B2M	7	0.188	10	1.046	10	0.705	10	1.053
	TUBB4	9	0.201	9	0.597	9	0.359	3	0.495
	TFRC	10	0.209	8	0.474	7	0.197	7	0.625
	RPS2	3	0.057	5	0.427	5	0.160	9	0.690
	RPL4	6	0.109	3	0.411	2	0.115	6	0.624
	ACTB	4	0.064	6	0.431	3	0.124	2	0.464
	GAPDH	8	0.191	7	0.455	8	0.217	4	0.560

*H* hypothalamus, *P* pituitary, *T* testis, *M* Average expression stability, *SV* Stability Value, *SD* Standard Deviation. With NormFinder and geNorm, low stability values indicate greater gene expression stability. With BestKeeper, genes with SD values greater than 1 are considered unstable.

**Figure 4:**
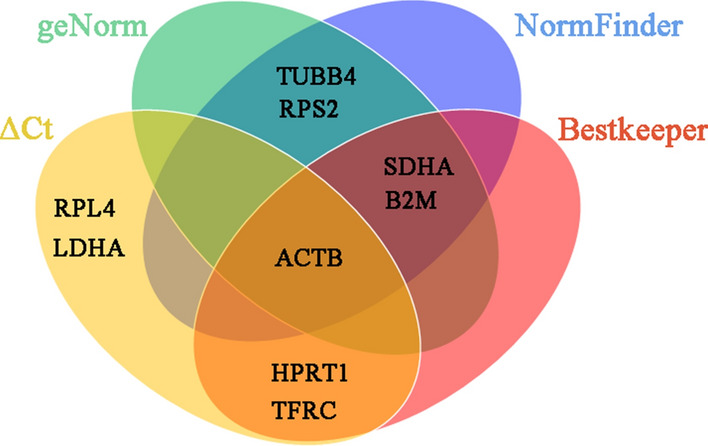
Venn diagrams of the top five internal reference genes derived from four software analyses of the entire reproductive axis.

In order to more intuitively show optimal reference genes applicable to the hypothalamus, pituitary and testis, we constructed Venn diagrams using the top five reference genes among the four analyses conducted (Fig. 4). Based on the four software analyses combined, *ACTB* was the most suitable reference gene of the whole reproductive axis of Magang geese subjected to different light treatments and different reproductive conditions.

### Determination of the optimal number of reference genes

**Figure 5:**
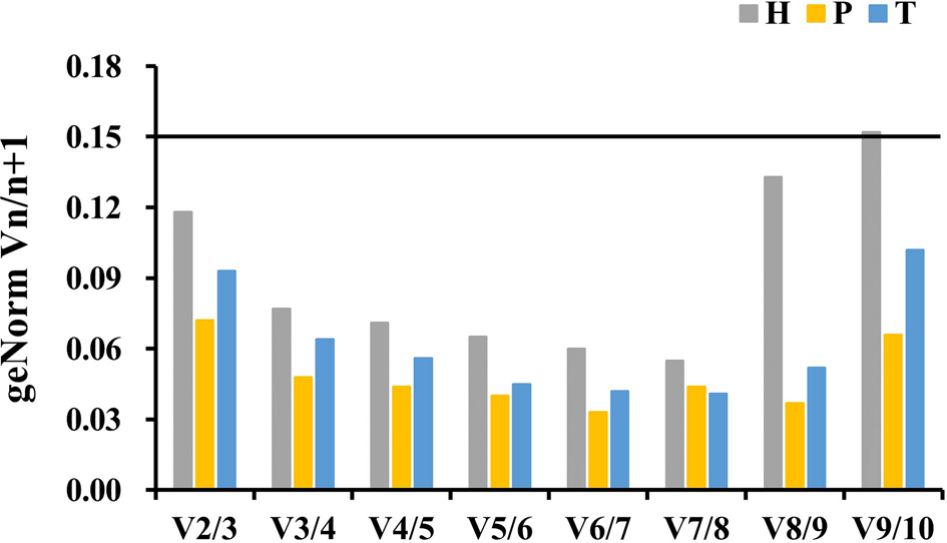
Determination of the optimal number of reference genes. *H* hypothalamus, *P* pituitary, *T* testis.

The geNorm program can also use standardized factors to analyze paired differences to evaluate the optimal number of required internal reference genes. The V value is the pairwise variation of the normalization factor after introducing a new internal reference gene. When Vn/Vn + 1 < 0.15, there is no need to introduce the next internal reference gene for correction, and the optimal number of internal reference genes is n. When Vn/Vn + 1 > 0.15, the next internal reference gene needs to be introduced for correction, and the optimal number of internal reference genes is n + 1^16,17^. The results showed that V2/V3 < 0.15 in the hypothalamus, pituitary and testis, and therefore did not need the introduction of a third reference gene. Thus, the optimal number of reference genes was two (Fig. 5). Combined with the stability analysis of the aforementioned reference genes, *ACTB* and *TUBB4*; *HPRT1* and *RPL4*; and *HPRT1* and *LDHA* can be used as optimal reference gene combinations in hypothalamic, pituitary, and testicular tissues during different reproductive stages.

### Evaluation and validation of selected reference genes

**Figure 6:**
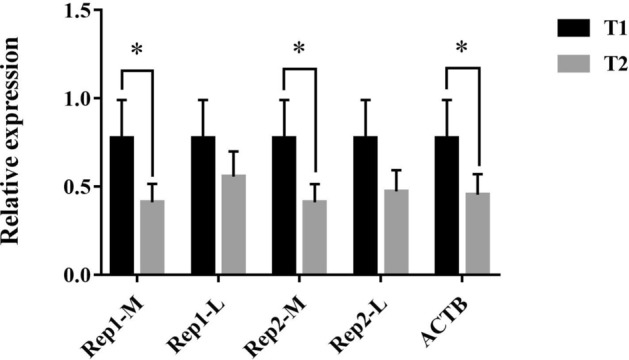
Validation of the selected reference genes by detected expression of the *FSHR* gene in the testis at different reproductive stages. *T1* reproduction period, *T2* reproduction decline period, *Rep1-M/L* the most stable (*HPRT1*) and least stable (*B2M*) reference genes, *Rep2-M/L* the two most stable (*HPRT1* and *LDHA*) and two least stable (*B2M* and *TFRC*) reference gene combinations; *P < 0.05.

To further verify the stability of the screened reference genes, two relatively stable reference genes (*HPRT1* and *LDHA*) and *ACTB* were selected to analyze the expression patterns of *FSHR* genes in testicular tissues of the breeding and reproductive decay periods. Moreover, the less stable reference genes (*B2M* and *TFRC*) were selected as controls. When normalizing a stable internal reference gene, the combination of two internal reference genes and *ACTB*, as well as the expression of *FSHR* in the testis tissue of the reproductive decline period was significantly lower (*P* < 0.05) than that of the breeding period (Fig. 6). When using internal reference genes with the least stability, no significant difference was noted in the expression of *FSHR* during the reproductive decline period and the breeding period (Fig. 6).

## Discussion

Quantitative PCR is one of the most powerful techniques used in the detection and determination of nucleic acid content in different types of samples. Therefore, it has a wide range of applications in the fields of life sciences, molecular diagnostics, agriculture and medicine^18^. Bustin^3^ pointed out that the selection of normalization genes is critical to the reliability of study findings. Lossos et al*.*^19^ showed that there is no single reference gene that can be applied to various cell types and different tissues. Therefore, it is necessary to screen suitable internal reference genes, especially for expression studies in some tissues that have been subjected to specific conditions.

It is generally believed that the amplification efficiency of qPCR between 90 and 110% is close to the ideal situation, and when the correlation coefficient R^2^ is greater than 0.98, there is a good correlation between the two values. Through calculation, we found that the amplification efficiency of the 10 candidate reference genes ranged from 92.845 to 107.615%, and the determination coefficient R2 ranged from 0.982 to 0.998 (Table 2), indicating that each reference gene had a good linear relationship within the concentration gradient of serial dilution, and the data was valid.

Studies have been conducted on the screening of reference genes in various tissues of geese. In various stages of granular cells of the Tianfu goose, the three most stable internal reference genes were *SDH*, *HMBS* and *18S*, and the three least stable were *UBC*, *GAPDH* and *TUB*^20^. In Zi geese, *GAPDH* was a relatively suitable internal reference gene for expression analysis of target genes during the egg laying stage and before the onset of lay. Ji et al*.*^21^ analyzed the stability of seven genes in five tissues of Zi geese and found that the expression of *HPRT1* was the most stable in muscle, 28S in the heart, *GAPDH* in the liver and ovaries, and *ACTB* in the kidney. In the current study, combining the results of four software analyses, we found that the optimal internal reference genes of various tissues of the Magang goose differ. The optimal internal reference gene combination in hypothalamic tissue is *ACTB* and *TUBB4*, and the optimal internal reference gene combination in pituitary tissue is *HPRT1* and *RPL4*. The most suitable internal reference gene combination in the testis is *HPRT1* and *LDHA*.

As stated in the minimum information for publication of quantitative real-time PCR experiments (MIQE) guidelines^3^, normalization of qPCR data using a single internal reference gene may lead to bias in the results; therefore, the use of two or more reference genes is recommended. The optimal number of required reference genes can be evaluated according to the geNorm procedure. If Vn/n + 1 is below the threshold of 0.15, the benefits of using additional (n + 1) reference genes will be limited. Previous studies have shown that the optimal number of reference genes required is two, in tissues of skeletal muscle, the spleen, and shell gland of chickens^12,22,23^. In the current study, the geNorm V value of the two most stably expressed reference genes in the hypothalamus, pituitary, and testis was < 0.15. Thus, the addition of a third stable gene was not necessary to normalize the expression data.

As stated in the MIOE guidelines^3^, normalization of qPCR data using a single internal reference gene may lead to bias in results, so it is recommended to use two or more reference genes. The optimal number of required reference genes can be evaluated according to geNorm procedure. Once Vn/n + 1 is below the threshold of 0.15, the benefits of using additional (n + 1) reference genes will be limited. Many studies found that the optimal number of required reference genes was two. Such as in skeletal muscle, spleen and shell gland of chickens^11,24^. In this study, the geNorm V of the two most stably expressed reference genes in hypothalamus, pituitary and testis was < 0.15, so it was not necessary to add a third stable gene to normalize the expression data.

Based on the analysis of the data from the three tissues, the *ACTB* gene ranked in the top five of the four software analyses, whereas *GAPDH* was found to have poor stability. *GAPDH* is involved in glycolytic pathways, and its expression depends on tissue type and specific conditions^22^, such as glucose deprivation and stress induction^25^. Recently, *GAPDH* has been found to be the most unstable reference gene in white adipose tissue and skeletal muscle in the inguinal adipose tissue of food-restricted mice^26^. In the current study, *GAPDH* ranked the highest with most statistical tools. Therefore, we suggest that *GAPDH* should not be used as a reference gene in geese subjected to light treatment.

*FSHR* was used as a target gene to validate our reference gene in the testis for qPCR. The combination of FSHR and FSH can promote Sertoli cells to produce inhibin and induce feedback inhibition of pituitary secretion and synthesis of FSH, and effectively stop spermatogenesis^27^. The reproductive characteristics of Magang goose are of short-day type. A lot of studies and practices have shown that long-day light can cause Magang goose to stop laying. We treated Magang geese with continuous long light (18L:6D), and the geese entered the rest period after 25 days of light treatment. Samples were taken before the initiation of light treatment (the breeding period); and at 14 days (breeding decay period); and 28 days (rest period) after light treatment. From the data analysis results, it can be seen that from the breeding period to the reproductive decline period, the expression level of *FSHR* in Magang geese should be significantly reduced. The two most stable and two most unstable internal reference genes were used for verification. The results showed that the most unstable gene significantly affected the results of normalization of expression data and might lead to misinterpretation of these data.

In summary, the optimal reference genes differed in various tissues of the reproductive axis of Magang geese subjected to light treatment. A combination of the two most stable reference genes was a more effective screening strategy. Use of the *ACTB* gene is recommended in the study of gene expression in tissues of the reproductive axis of Magang geese subjected to various light treatments.

## Methods

### Ethics statement

The experimental setup was approved by the Animal Care Committee of the Zhongkai University of Agriculture and Engineering in China. All applicable institutional and governmental regulations concerning the ethical use of animals were followed, and all efforts were made to minimize animal suffering.

### Animals

The study was conducted at a goose farm in Qingyuan, Guangdong province from October 1 to November 1, 2019. A total of 420 (male:female = 1:5) 3-year-old Magang geese during the normal breeding period were selected. The geese were exposed to natural light (13L:11D) before the study commenced, and were then treated with light on October 1. The duration of light was gradually extended to 18L:6D after October 3, and the geese entered the rest period after 25 days of light treatment. Eight male geese were randomly selected one day before the initiation of light treatment (the breeding period); and at 14 days (breeding decay period); and 28 days (rest period) after light treatment. The experimental animals were humanely euthanized, and the hypothalamic, pituitary, and testis tissues were collected. The tissue samples were rinsed with diethylpyrocarbonate (DEPC) water and put into a cryopreservation tube, and then quickly placed in a liquid nitrogen tank for cryopreservation, and stored at − 80 °C.

### RNA isolation and cDNA synthesis

The total RNA of the goose hypothalamus, pituitary and testis was extracted by the magnetic bead method (Xi'an Tianlong Technology, China), and the concentration and purity of total RNA were detected using an Epoch microplate spectrophotometer (BioTek, USA). The RNA integrity was determined by 1.0% agar-gel electrophoresis. The cDNA was synthesized using the ReverTra Ace qPCR RT Master Mix with gDNA Remover (Code No. FSQ-301), following the recommended manufacturer’s protocol (TOYOB, Shanghai) and stored at − 20 °C.

### Reference gene selection and primers optimization

A total of 10 genes (*HPRT1, GAPDH, ACTB, LDHA, SDHA, B2M, TUBB4, TFRC, RPS2*, and *RPL4*) were selected as candidate internal reference genes for analysis of expression stability. Reference genes were chosen based on the literature, and nucleotide sequences were recovered from GenBank (www.ncbi.nlm.nih.gov/genbank/). All primers were designed using the Premier Primer 5.0 software (Premier Bio-soft International, CA, USA) and synthesized by Sangon Biotech Co. Ltd (Shanghai, China). Primer BLAST was used for detection to confirm the specificity of primer sequences, and primer dimers and hairpin structures were verified. The primer data of the candidate internal reference genes are presented in Table 3.

**Table 3:** Primer information of candidate internal reference genes and target genes.

No.	Gene symbol	Gene name	Accession no.	Primer sequence (5ʹ–3ʹ)
1	HPRT1	Hypoxanthine phosphoribosyltransferase	AJ132697	F: GCACTATGACTCTACCGACTATTG R: CAGTTCTGGGTTGATGAGGTT
2	LDHA	Lactatedehydro-genase A	ENSGALE00000067556	F: CTATGTGGCCTGGAAGATCAG R: GCAGCTCAGAGGATGGATG
3	SDHA	Succinate dehydrogenase complex, subunit A	XM_013195307.1	F: AAAAGGAGGACAGGCTCACA R: ACACCACGACACTCTCCATT
4	B2M	Beta-2-microglobulin	XM_013198887.1	F: GTCCTCAACTGCTACGTGGA R: AGGTGTAGACGTCGCTCTTG
5	TUBB4	Tubulin beta-4 chain	XM_013182843.1	F: CTGGCAGTCAACATGGTTCC R: ACATACGGCCCCTAAACACA
6	TFRC	Transferrin receptor (p90, CD71)	XM_013195023.1	F: GAAGTGGCAAGTGTGAGGTG R: ATCTTCACTCTGGCCAGCTT
7	RPS2	Ribosomal protein S2	XM_013174975.1	F: TCCCAAGAAGCTGCTGATGA R: TCTGCACTGAGACTCTGGTG
8	RPL4	Ribosomal protein L4	XM_013193267.1	F: GCAAACCCGCTACTCTGAAG R: TTTTGGCGTACGGGTTCAAC
9	ACTB	β-Actin	M26111	F: CAACGAGCGGTTCAGGTGT R: TGGAGTTGAAGGTGGTCTCG
10	GAPDH	Glyceraldehyde-3-phosphate dehydrogenase	DQ821717.1	F: GCTGATGCTCCCATGTTCGTGAT R: GTGGTGCAAGAGGCATTGCTGAC
11	VIP	Vasoactive intestinal peptide	XM_013178196.1	F: TTGATGCAGCCAGTGAACCT R: TTCCGAAAGCGGCTGTAGTT
12	FSHR	Follicle stimulating hormone receptor	XM_013192472.1	F: GATGAGCAACCTGGCAATAAG R: GGTGAGCAAGCCACATTAAC

Each primer pair was subjected to the PCR and then detected by 1.5% agarose gel electrophoresis and DNA sanger sequencing. The PowerUp SYBR Green Master Mix fluorescent quantitative kit (Invitrogen, CA, USA) and QuantStudio 7 Flex real-time PCR detection system (Applied Biosystems, CA, USA) were used for quantitative analysis of the RT-qPCR. The cDNA template was diluted into six gradients (1, 10^–1^, 10^–2^, 10^–3^, 10^–4^, and 10^–5^), and qPCR detection was performed with these serial gradient concentrations of the cDNA template.

The following experimental RT-qPCR conditions were used: one cycle at 95 °C for 10 min; 40 cycles at 95 °C for 10 s; and 60 s at 60 °C–62 °C. Additional steps with a gradual increase in temperature from 60 to 62 °C up to 95 °C were used to obtain a dissociation curve. The data were analyzed using the ABI 7500 software v2.3 software to generate standard curves and melting curves of the genes. The following formula E = (10^–1^/slope − 1) × 100 was used to calculate the PCR amplification efficiency of each target gene and reference gene^3,28,29^.

### Quantitative real-time PCR

The expression of 10 genes in different tissues of eight individual geese at each stage were analyzed using qPCR with a 20 µL reaction system as follows: SYBR Master Mix 10 µL; ddH2O 8.6 µL; upstream and downstream primers each, 0.2 µL; and cDNA template, 1 µL. Each sample was assayed in triplicate under the following conditions: The reaction conditions were as follows: pre-denaturation at 95 °C for 30 s; denaturation at 95 °C for 5 s; annealing at 60 °C; extension for 30 s, over 40 cycles.

### Determination of expression stability of reference genes

The stability of candidate reference genes was comprehensively evaluated using four methods: ΔCT, geNorm, NormFinder, and BestKeeper^8–10,30^. The relative expression level was calculated by the 2^−ΔCt^ method (ΔCt = Ct, sample—Ct, min). The gene showing the least expression among all the samples was first identified, and its Ct value (highest expression quantity) was determined. That lowest Ct value was then subtracted from the Ct value of a given sample gene, and the obtained ΔCt value was typically 0 or higher. After obtaining the ΔCt value of each gene of each sample, the Excel 2003 software was used to calculate the 2^−ΔCt^ value of each corresponding gene for each corresponding sample. This value represented the relative quantitative data of each candidate internal reference gene, which was also used in the analysis of geNorm software data.

GeNorm software analysis was first used to calculate the relative quantitative data of each reference gene by the 2^−ΔCt^ method. A table constructed with the Excel software was then imported into the geNorm program for further calculations. The M values of stability were derived for each internal reference gene, to determine the more suitable gene. Those with values lower than 1.5 were considered stable genes^31,32^. The calculation methods of the NormFinder program analysis were similar to those of the geNorm program. Both are based on the 2^−ΔCt^ method to calculate the relative quantitative data of internal reference genes. The BestKeeper program used a built-in formula to calculate the SD of the pairing between each reference gene. Judgment criteria were as follows: the smaller the SD value, the better the stability of the internal reference gene, and if the SD > 1, the gene was considered unstable^33,34^. The ΔCT method was used to calculate the average SD of each gene to determine the stability of the internal reference gene. The smaller the s value, the higher the stability of the gene. As each method is based on different algorithms, the results obtained may also differ.

In order to avoid any one-sidedness in the use of a single evaluation method, multiple evaluation methods can be combined for more comprehensive evaluations^35^. In the current study, the geometric mean of the evaluation rankings obtained by the four analysis methods was derived, and the stability of the reference genes was determined by the final comprehensive ranking index. The smaller the index, the more stable the expression of the reference genes.

### Reference gene validation

In order to further verify the stability of the internal reference genes obtained through screening, the two most stable internal reference genes were selected to analyze the expression patterns of *FSHR* genes in tissues of the reproductive axis of Magang goose during different reproductive stages (breeding period, reproductive decline period, and rest period). Moreover, the most unstable internal reference genes were selected as the control group.
